# Anti-Inflammatory, Antioxidant, and Healing-Promoting Effects of *Aloe vera* Extract in the Experimental Colitis in Rats

**DOI:** 10.1155/2021/9945244

**Published:** 2021-12-06

**Authors:** Mahvash Alizade Naini, Asal Zargari-Samadnejad, Shayan Mehrvarz, Romina Tanideh, Mohammad Ghorbani, Amirreza Dehghanian, Maryam Hasanzarrini, Farnaz Banaee, Omid Koohi-Hosseinabadi, Nader Tanideh, Aida Iraji

**Affiliations:** ^1^Department of Internal Medicine, School of Medicine, Shiraz University of Medical Sciences, Shiraz, Iran; ^2^Gastroenterhepatology Research Center, Shiraz University of Medical Sciences, Shiraz, Iran; ^3^Zarmehr Darou Danesh Knowledge Based Company, Pharmaceutical Products Technology Incubator, Shiraz University of Medical Sciences, Shiraz, Iran; ^4^Student Research Committee, Shiraz University of Medical Sciences, Shiraz, Iran; ^5^Department of Public Health, School of Health, Torbat Heydariyeh University of Medical Sciences, Torbat Heydariyeh, Iran; ^6^Health Sciences Research Center, School of Health, Torbat Heydariyeh University of Medical Sciences, Torbat Heydariyeh, Iran; ^7^Trauma Research Center, Shiraz University of Medical Sciences, Shiraz, Iran; ^8^Department of Pathology, Shiraz University of Medical Sciences, Shiraz, Iran; ^9^Department of Gastroenterology, School of Medicine, Hamadan University of Medical Sciences, Hamadan, Iran; ^10^Laparoscopy Research Center, Shiraz University of Medical Sciences, Shiraz, Iran; ^11^Central Research Laboratory, Shiraz University of Medical Sciences, Shiraz, Iran; ^12^Stem Cells Technology Research Center, Shiraz University of Medical Sciences, Shiraz, Iran; ^13^Department of Pharmacology, School of Medicine, Shiraz University of Medical Sciences, Shiraz, Iran

## Abstract

**Background:**

Ulcerative colitis is a worldwide chronic gastrointestinal disease characterized by variable extensions of colon mucosal inflammation. The available drugs have an incomplete response with various side effects and socioeconomic impacts. *Aloe barbadensis Miller (Aloe vera)* is a well-known medicinal plant with diverse pharmacological and therapeutic activities. As a result, in the current study, *Aloe vera* was selected to evaluate its therapeutic effects on experimental colitis in rats.

**Methods:**

This study is intended to evaluate the possible beneficial effect of *Aloe vera* for the treatment of experimental colitis. Trinitrobenzenesulfonic acid (TNBS) was used to induce experimental colitis in 60 of 70 Wistar rats. The rats were grouped in 7 clusters including healthy control, negative, positive control (received sulfasalazine), and test groups treated with *Aloe vera* extracts *via* oral or rectal routes. Macroscopic and histologic factors as well as the biochemical parameters were evaluated on day 7.

**Results:**

In the present study, it was found that serum levels of tumor necrosis factor-*α* (75 vs. 44 pg./ml), interleukin-6 (41 vs. 21 pg/ml), and nitric oxide (24 vs. 6 *μ*m/ml) in TNBS-induced untreated colitis treatment were significantly increased as compared to healthy control. Similar patterns were also observed in malondialdehyde (76.41 vs. 236.35 *μ*g/mg) and myeloperoxidase (4.24 vs. 29.38 U/mg) in colonic tissue. Among different treatments, rectal administration of *Aloe vera* extract (400 mg/kg) exhibited the best result in which serum concentration of tumor necrosis factor-*α* (55 pg/ml), interleukin-6 (24 pg/ml), and nitric oxide (10 *μ*m/ml) and the levels of malondialdehyde (102.67 *μ*g/mg), as well as myeloperoxidase (12.29 U/mg) in colon tissue, were reduced as compared to the untreated group. Also, the body weight and colon weight/length ratios were more improved in the treated group with 400 mg/kg *Aloe vera* extract, rectally.

**Conclusion:**

*Aloe vera* extract exhibited a therapeutic effect in TNBS-induced colitis, and local, rectal administration of *Aloe vera* extract was more effective than oral administration.

## 1. Introduction

Ulcerative colitis (UC) as an annoying chronic problem is one of the two major subtypes of inflammatory bowel diseases (IBDs) with different geographic prevalences and worldwide distribution [1, 2]. Although UC may present insidiously, its hallmark is subacute bloody diarrhea, accompanied by anemia and fatigue. It also may change to acute severe colitis, presenting temperature above 37.5°C, heart rate above 90/min, and hemoglobin concentration below 10.5 g/dL with more than 6 bloody stools daily [3, 4].

Its manifestation is due to continuous inflammation of the rectum with the variable extension but usually with decreasing severity up to the cecum [5]. Its etiology and exact underlying pathophysiologic aspects are unclear, but most probably is due to aberrant deregulated mucosal immune responses (humoral and cellular immunity) to environmental factors in a genetically susceptible population. Following epithelial barrier dysfunction and immune cell activation, inflammatory cytokines and mediators (interferon-*γ* (IFN-*γ*), interleukin-2 (IL-2), IL-4, IL-5, IL-10, IL-13, IL-17, IL-23, and tumor necrosis factor *α* (TNF-*α*)) are released which may be used as disease activity indicators [6, 7]. The diagnosis of UC is based on clinical presentation and chronic colon inflammation confirmed by histology [8]. Uncertain definitive pathogenesis, variable presentation, natural course, and lack of standard disease activity index are obstacles for definite therapeutic effect assessment. In any case, the accepted therapeutic goals are (a) accentuating induction of remission and maintenance period, (b) improving the nutritional status, (c) decreasing disease complications, and (d) considering side effects and cost effectiveness. In current medicine, the main treatments are focused on 5-ASA and steroids. Biologic therapies such as antitumor necrosis factor antibodies are prescribed for resistant patients. Antiadhesion molecules and kinase inhibitors are under research for UC treatment [9–12]. The inadequate response, frequent relapse, steroid dependency, and side effects result in developing a new candidate as the second line of treatment. Considering therapeutic effects of some herbal medicine such as heartleaf houttuynia [13, 14], boswellic acid [15, 16], diamonnium glycyrrizhinate [17], slippery elm [18], fenugreek [18], devil's claw [18], tormentil [18], and wei tong ning [18] in various diseases, especially in China, Middle-East, and Africa, new research in this field is rational.

*Aloe vera* (*Aloe barbadensis* Mill.) belongs to the Aloeaceae family with thick, tapered, green lance-shaped, juicy, sharp, and edged leaves [19]. *Aloe vera* grows in dry regions of Africa, Europe, Asia, and America. *Aloe vera* is probably the most applied medicinal plant commercially and the most popular plant worldwide [20]. Various parts of the plant contain amino acids, sugars, enzymes, vitamins, minerals, saponins, anthraquinones, lignin, and salicylic acid. Also, the leaves are the source of various organic acids, phenolic compounds, minerals, and vitamins [21]. Therapeutic effects of *Aloe vera* in wound healing [22], inflammation, intestinal absorption, and reducing oxidative status were assessed in recent research [23]. It also has been used empirically to increase high-density lipoprotein, reduce low-density lipoprotein, and decrease glycemia in diabetics [19]. Furthermore, the anti-inflammatory effects of *Aloe vera* in the human colon were confirmed *in vitro* by Langmead et al. [24]. In 2017, the healing effect of the aqueous leaf extract of *Aloe vera* in an animal model of experimentally induced colitis was investigated. The favorable effects confirmed through the significant reduction in Bax mRNA expression and elevation in Bcl-2 mRNA expression when compared with the colitis group without treatment [25]. In another study, 50 and 300 mg/kg of *Aloe vera* gel extract were used to evaluate the improvement in the symptoms of UC in rats. According to microscopy and macroscopic observations, the symptoms of UC were improved significantly [26]. Park et al. showed that 0.1% and 0.5% aloesin supplement (one of the compounds of *Aloe vera*) decreased the myeloperoxidase (MPO) activities as well as TNF-*α* and interleukin-1*β* (IL-1*β*) mRNA expressions on the UC rat colitis model [27]. In another study, glucomannan extracted from *Aloe vera* balanced pro- and anti-inflammatory cytokines regulated the expressions of TLR-2 and improved the health state of colitis in mice [28]. Similarly, assessments on polysaccharides extracted from *Aloe vera on* UC-animal models depicted an improvement in colitis, *via* JAK2, p-JAK2, STAT-3, and p-STAT3 protein expression [29]. In a randomized, double-blind, placebo-controlled trial, oral *Aloe vera* gel (100 mL twice daily for 4 weeks, in a 2 : 1 ratio) was administered for active UC patients. The supplement reduced the clinical colitis activity index and histological scores significantly during treatment with *Aloe vera* [30].

However, it seems that further evaluation about the therapeutic potential of *Aloe vera* extract on UC as well as its effect on new biochemical factors related to UC is needed to get more insight into signaling pathways. Furthermore, in the current study, for the first time, the different routes and doses of *Aloe vera* administrations (intragastrically and rectally) were studied. Regarding the therapeutic dose of *Aloe vera* used in the previous studies with no report of toxicity in the tested range, 200 and 400 mg/kg *Aloe vera* extract were chosen for further study [31–35]. This study was designed to evaluate and compare the dose and route treatment of *Aloe vera* extract on colitis in rats and its impacts on proinflammatory cytokines.

## 2. Materials and Methods

### 2.1. Ethical Statement

The animal experiments were performed in accordance with the guidelines of the Laboratory Animal Center of Shiraz Medical University (No. 91-01-36-4560). All the experimental procedures were strictly conducted according to the international standards and national legislation on animal care and the Animal Research Reporting In Vivo Experiments (ARRIVE) guidelines. Experimental research on the plant was under international legislation and guidelines of the Pharmacognosy Department of Shiraz University of Medical Sciences, Shiraz, Iran. At the end of the study, rats were euthanized with the rapid and humane method using a 70% volume displacement rate of CO_2_ increased to around 100% in the induction chamber.

### 2.2. Study Design and Induction of Colitis

The Laboratory Animal Center of Shiraz University of Medical Sciences with a pathogen-free environment, constant temperature (23 ± 2), and acceptable humidity (55 ± 5%) provided us with 70 male Wistar rats (175–215 grams) supplied with a balanced diet along with free access to water. The rats were fasted with free access to water for 24 h before induction of colitis. After rats were anesthetized with ketamine (50 mg/kg i.p), the rubber-tipped gavage needle was inserted into the anus of rats (7 cm) and 1 ml solution of 2,4,6-trinitrobenzenesulfonic acid (TNBS, 150 mg/kg dissolved in ethanol) was slowly injected into the colon while the control group received only ethanol. Animals were held in the head-down position for 30 seconds and then returned to their cages [36–38]. Later, water and food were available. 12 hours after colitis induction, the treatments were started and continued one a day for six consecutive days. The effectiveness of treatment was assessed by clinical, macroscopic, biochemical, and histopathological assessments. The rats' general conditions were assessed daily.

### 2.3. Experimental Animals

A total of 70 Sprague Dawley male rats (aged 10-12 weeks, weighing initially 220 ± 20 gram) were obtained from the Laboratory Animal Center of Shiraz University of Medical Sciences. Animals were divided into seven groups (10 rats per group, *n* = 10).

  Group (1): healthy control group
  Group (2): TNBS-induced colitis untreated rats
  Groups (3): TNBS-induced colitis treated rats who received 200 mg/kg *Aloe vera* extract once a day, intragastrically
  Groups (4): TNBS-induced colitis treated rats who received 400 mg/kg *Aloe vera* extract once a day, intragastrically
  Groups (5): TNBS-induced colitis treated rats who received 200 mg/kg *Aloe vera* extract once a day, rectally
  Groups (6): TNBS-induced colitis treated rats who received 400 mg/kg *Aloe vera* extract once a day, rectally
  Group (7): TNBS-induced colitis treated rats who received 500 mg/kg sulfasalazine once a day, intragastrically as a positive control group

The dose of *Aloe vera* extract for treatments was selected according to previously reported research [33–35]. According to the published articles, evaluation on the acute and subacute toxicity of *Aloe vera* in rats indicated that the methanol extract at the doses of 1, 2,4, 8, and 16 g/kg B.wt did not produce significant toxic effects [31]. In the other study, assessments on the subacute toxicity test showed that *Aloe vera* did not produce marked subacute toxic effects up to a maximum concentration of 3330 mg/kg body weight on rats with no mutagenic activity in ICR mice exposed to 10000 mg *Aloe vera*/kg body weight [32]. As a result, at the tested dose of *Aloe vera* extract, the toxic effect in rats without colitis was not assessed.

Sulfasalazine also was purchased from Merck chemical company. The appropriate amount of extract or sulfasalazine based on the treated group was dissolved in sterile water. Intragastric administration was used in conscious rats with biomedical needles (length 76.2 mm, diameter 3 mm, straight). To prepare extract for rectal administration, 5% glycerol was mixed with 2% sodium carboxymethyl cellulose (NaCMC) as an inert preservative substance [39]. Next, 200 and 400 mg/kg body weight of the dried extract was dissolved in deionized water, and the mixtures were gradually added to the glycerol-NaCMC solution. The gel was homogenized for 30 minutes, and the gel was collected in an aluminum tube in the refrigerator. For rectal administration, the gavage needle was inserted into the anus of rats (7 cm) and 1 ml of the prepared gel was injected [40].

### 2.4. Plant Extract

*Aloe vera* leaves were obtained in Shiraz, Fars Province, Iran, and its species was endorsed by SUMS taxonomists at a pharmacy school. 100 g dried *Aloe vera* was powdered and percolated with 70% ethanol (3 times), at room temperature, and the extracts were filtered and evaporated under reduced pressure to acquire 9.8 g of dried extracts (9.8% yield). This procedure was repeated several times to get enough amounts of extract for in vitro and in vivo studies.

### 2.5. Macroscopic Scoring

The dosage and period of treatments were accompanied by daily body weights, gross stool evaluation for visible and/or occult bleeding. On the last day of the experiment (7^th^), the degree of colonic inflammation and damage was scored (Table 1) as described by Morris et al. with slight modifications [41, 42].

### 2.6. Histopathological Scoring

After the 10 cm of distal colon segments of slaughtered rats have been dissected (under the guide of anesthesia), flushed with cool saline, and weighted, histologic assessment of colitis was performed by a drug-blinded expert pathologist in SUMS. The histopathological assessment of damage was determined according to the numerical grading score introduced by Wallace et.al outlined in Table 2 [43]. The edema intensity was estimated by calculating the ratio of wet tissue weight to the length of the colon.

### 2.7. TNF-*α*, IL-6, and NO

In the last days of research, blood samples were collected and centrifuged at 4000 rpm for 20 min; the serum cytokines including TNF-*α* (TNF-alpha assay kit, Diaclone, Besançon cedex, France), IL-6 (Interleukin-6 assay kit, ZellBio GmbH, Ulm, Germany), and NO (Nitric oxide assay kit, KNO-96, KiaZist, Bahar, Hamadan, Iran) were determined [44–46].

### 2.8. Assessment of Colonic MPO

The tissue of the colon was measured and weighted, ripped, and homogenized in 10 ml of cold 50 mmol potassium phosphate buffer (pH of 6.0), containing 0.5% hexadecyltrimethyl ammonium bromide (HETAB) and 10 mmol ethylenediaminetetraacetic acid (EDTA) [47]. The homogenates were centrifuged at 2000 rpm for 20 min; MPO was assessed in the supernatant liquid (MPO activity kit, KMPO-96, KiaZist, Bahar, Hamadan, Iran).

### 2.9. Assessment of Malondialdehyde (MDA)

MDA is the main end product of polyunsaturated fatty acids oxidation, so its concentration (nmol/g wet tissue) indicates the extent of lipid peroxidation. MDA level of the tissue was measured with an MDA assay kit (Lipid peroxidation kit, KMDA-96, KiaZist, Bahar, Hamadan, Iran). Briefly, 20 mg of colon tissue samples were rinsed with phosphate-buffered saline (PBS) and then were homogenized in 300 *μ*L of MDA lysis buffer containing 3 *μ*L of butylated hydroxytoluene (BHT) on ice. The resulting solution was centrifuged for 10 minutes at 6000 × g, and the supernatant was separated for further evaluation. To 600 *μ*L of (thiobarbituric acid) TBA solution, a 200 *μ*L sample was added and the mixture was incubated at 95°C for 60 minutes. After cooling to room temperature, 200 *μ*L of the mentioned mixture was added to a 96-well plate and the absorbance was measured spectrophotometrically at 532 nm [48–50].

### 2.10. Statistical Analysis

Statistical analysis was performed with SPSS statistical software (Version 19.0, Chicago, IL, USA). Results are expressed as mean ± standard error of the mean (SEM). Kolmogorov–Smirnov tests were used for the assessment of normality. Analysis of variance (ANOVA) followed by Tukey's multiple comparison post hoc test was conducted. *P* values less than 0.05 were considered statistically significant.

## 3. Results

### 3.1. Effects of *Aloe vera* Extracts on General Condition, Body Weight, and Colon Weight/Length

**Figure 1:**
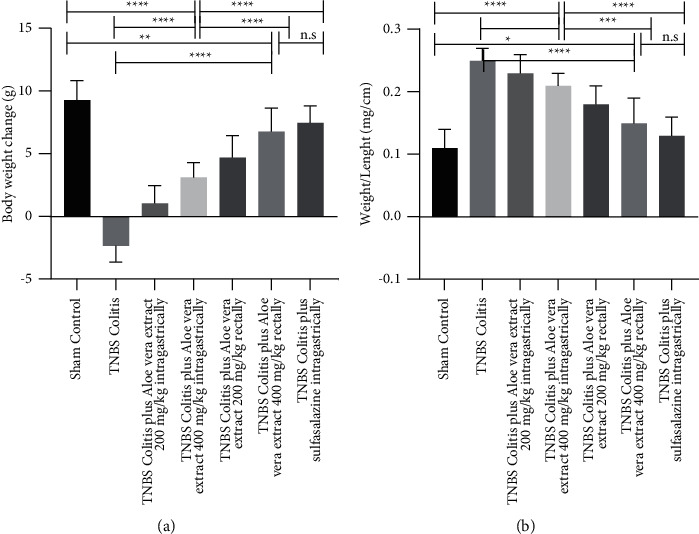
Effects of trinitrobenzenesulfonic acid- (TNBS-) induced colitis and administration of *Aloe vera* extract or sulfasalazine on body weight change in comparison to the initial body weight before the start of the study (a) and the ratio of colon weight to colon length (b) at the 7^th^ day after induction of colitis. Data are expressed as mean ± SD. *N* = 10 in each experimental group. n.s. = not statistically significant. ^*∗*^*P* value <0.05; ^*∗∗*^*P* value <0.01; and ^*∗∗∗∗*^*P* value <0.0001.

TNBS-induced rats had hypomotility, frequent loose, purulent, and bloody stools with significant weight loss compared to the sham control group. The rats treated with *Aloe vera* extracts in different doses (200 mg or 400 mg/kg) *via* different routes (intragastrically or rectally) and sulfasalazine (500 mg/kg) showed remarkable dose- and route-dependent improvement of mentioned symptoms after 7 days of treatment. The body weight changes and improved colon weight/length ratios are demonstrated in Figure 1 on the 7^th^ day of research.

### 3.2. Effects of *Aloe vera* on Macroscopic Changes in TNBS-Induced Colitis

The gross semblance of the untreated TNBS-induced colon model was severe edema, inflammation, and hyperemia compared with the healthy group that had no inflammation. As shown in Table 3, the administration of *Aloe vera* extracts and sulfasalazine significantly improved the macroscopic scores of colitis with decreasing hyperemia and inflammation of the colon in TNBS-induced groups. These improvements were dose and route dependent. The dose of 400 mg *Aloe vera*/kg *via* the rectal route had the highest therapeutic effect which was comparable to sulfasalazine.

### 3.3. Effects of *Aloe vera* on Histologic Changes in TNBS-Induced Colitis

**Figure 2:**
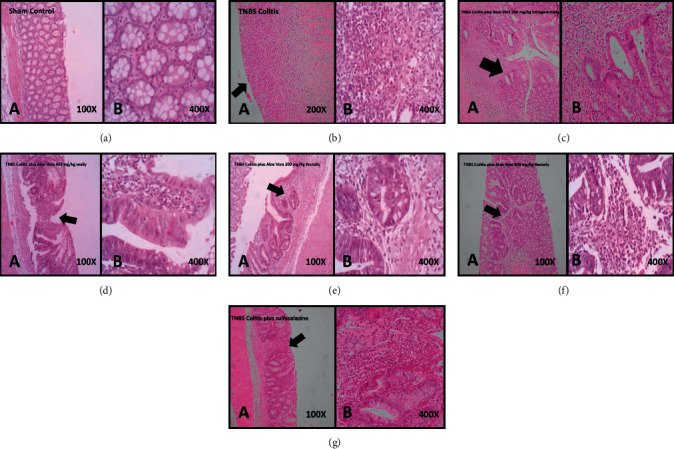
Histopathological colonic mucosal sections at different groups contain ten rats (*n* = 10): sham control mucosa with the intact epithelial surface and crypts ((a)- A, B), TNBS-induced colitis with severe mucosal ulceration, and totally glandular destruction, as well as crypt abscess formation and loss of mucosal architecture (arrow) ((b)- A, B, X100, X400), TNBS colitis treated with *Aloe vera* extract 200 mg/kg intragastrically shows moderate loss of mucosal architecture, cryptitis (arrow), goblet cell depletion, and muscle thickening ((c)- A, B, X100, X400), TNBS colitis treated with *Aloe vera* extract 400 mg/kg intragastrically shows mild loss of mucosal architecture (arrow), cryptitis, goblet cell depletion, and muscle thickening ((d)- A, B, X100, X400), TNBS colitis plus *Aloe vera* extract 200 mg/kg rectally shows mild loss of mucosal architecture, cryptitis (arrow), goblet cell depletion, and muscle thickening ((e)- A, B, X100, X400), TNBS colitis plus *Aloe vera* extract 400 mg/kg rectally shows mild cryptitis, goblet cell depletion (arrow), and muscle thickening ((f)- A, B, X100, X400), and TNBS colitis treated with sulfasalazine ((g)- A, B, X100, X400) with mild glandular destruction (arrow), cryptitis, and goblet cell depletion.

The histologic appearance of untreated TNBS-induced colitis mucosa was edema, ulceration, acute inflammatory cell infiltration, loss of cells, distorted mucosal architecture, and thickening of lamina propria (Table 3 and Figure 2). The extent and severity of inflammatory cell infiltration and cellular damage were significantly attenuated by treatment with *Aloe vera* extracts rectally and intragastrically (200 and 400 mg/kg) and sulfasalazine (500 mg/kg). These treatments also improved edema of the colon mucosa and crypt architecture. The changes were dose and route dependent, so more improvement was seen with 400 mg/kg *Aloe vera* extract through the rectal route and sulfasalazine, intragastrically.

### 3.4. Effects of *Aloe vera* Extract on the Secretion of TNF-*α*, IL-6, and NO in Rat Serum

**Figure 3:**
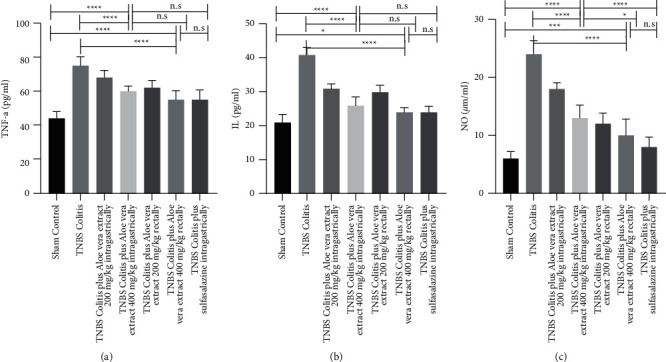
Effects of trinitrobenzenesulfonic acid- (TNBS-) induced colitis and administration of *Aloe vera* extract or sulfasalazine on the serum concentration of tumor necrosis factor-*α* (TNF-*α*) (a); serum concentration of interleukin-6 (IL-6) (b); and serum concentration of nitric oxide (NO) (c) at the 7^th^ day after induction of colitis. Data are expressed as mean ± SD. *N* = 10 in each experimental group. n.s. = not statistically significant. ^*∗*^*P* value <0.05; ^*∗∗∗*^*P* value <0.001; and ^*∗∗∗∗*^*P* value <0.0001.

By induction of colitis, as shown in Figure 3, the TNBS group had significantly (*P* ≤ 0.00001) higher mean serum levels of inflammatory cytokines, TNF-*α* (44 vs.75 pg./ml), IL-6 (21 vs.41 pg./ml), and NO (6 vs. 24 *μ*m/ml), compared to healthy control rats. Seven days of *Aloe vera* extract and sulfasalazine administration showed the reduction in the serum level of these cytokines, suggesting the inhibitory effect of treatments on proinflammatory cytokines. The significant (*P* ≤ 0.001 for all) anti-inflammatory effects of the *Aloe vera* extracts were dose and route dependent with more reduction with 400 mg rectally which was 51 pg./ml for TNF-*α*; 24 pg./ml for IL6; and 9 *μ*m/mL for NO.

### 3.5. Effects of *Aloe vera* Extract on MPO

**Figure 4:**
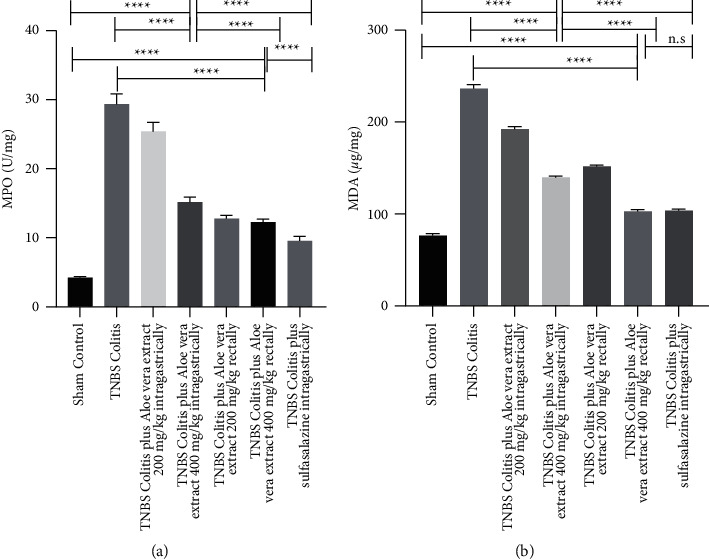
Effects of trinitrobenzenesulfonic acid- (TNBS-) induced colitis and administration of *Aloe vera* extract or sulfasalazine on colon tissue of myeloperoxidase activity (a); malondialdehyde level (b) at the 7^th^ day after induction of colitis. Data are expressed as mean ± SD. *N* = 10 in each experimental group. n.s. = not statistically significant. ^*∗∗∗∗*^*P* value <0.0001.

The MPO activity as evidence for inflammation was measured, and the results are reported in Figure 4(a). The mean level of MPO activity was significantly higher in the TNBS-induced colitis group compared with the control group (30.52 vs. 4.08 U/mg, *P* < 0.0001). The MPO activity decreased in all treated groups, especially in the 400 mg/kg *Aloe vera*-treated group rectally. However, none of the *Aloe vera*-treated groups achieved sulfasalazine decrement level.

### 3.6. Effects of *Aloe vera* Extract on Cell Lipid Peroxidation

As shown in Figure 4(b), the MDA level increased significantly in the untreated TNBS-induced colitis group as compared with the sham control group (241.05 vs. 73.77 *μ*g/mg wet colon tissue, *P* < 0.001). The levels decreased significantly in all treated groups. The decrements were dose and route dependent in *Aloe vera*-treated groups; however, 400 mg/kg administrated rectally had nearly the same effect as sulfasalazine (102.67 vs.103.52 *μ*g/mg).

## 4. Discussion

UC is a major gastrointestinal problem, with high impacts on health, and its socioeconomic burdens mandate proper consideration. However, commercially available drugs have lots of disadvantages including limited response and accessibility, as well as economic problems in developing countries. Based on recent cohort studies, although with the variable in results, UC may exceed up to 83% in 10 years' follow-up confirming not only the importance of UC but also the need to find a new treatment for UC in the near future [51, 52]. In this manner, more research in alternative medicines such as probiotics, dietary fibers, and herbal products has been suggested [53–56].

UC mostly affects the mucosal lining of the colon and rectum so that the infiltration of leukocytes and inflammatory cells, especially neutrophils, in addition to the overproduction of proinflammatory cytokines such as TNF-a, IL-1*β*, and prostaglandins resulted in mucosal disruption and ulceration [57]. MPO as a cytoplasmic enzyme protects the cell contents against oxidizing activity by destroying hydrogen peroxide (H_2_O_2_). MPO is known as one of the indicators of inflammation, and it is well correlated with leukocyte adhesion and accumulation in the intestine [58]. Oxidative stress is believed to be a key factor in the pathogenesis of UC. In detail, ROS, RNS, and oxygen peroxide increase lipid peroxidation, enzymatic dysfunction, and DNA or/and RNA strand breaking, as well as protein impairment that gives rise to oxidative stress and cell death [59–61]. Uncontrolled lipid peroxidation in UC is accompanied by the formation of a complex mixture of products including aldehydes, especially toxic MDA [62]. Lots of research studies express that penetration of the mucosa by T cells and the increased production of proinflammatory cytokines are hallmarks of IBD. It is well documented that blood and plasma levels of TNF-*α*, IL-6, and NO were raised in patients having UC [63]. TNF-*α* is not only an important component in the pathophysiology of UC but also extends inflammation by activating NF-*κβ* pathways, which contribute to ulceration and degradation of the mucosa through the release of matrix metalloproteinases (MMPs). IL‐6 is another predominant cytokine found in inflamed areas from UC patients, and the other predominant cytokines were related to a Th1 profile. Different studies confirmed that induced UC in rats increases immunomodulator indexes confirming that oxidative stress could be correlated to disease [36, 64–67]. Under normal conditions, ROS is naturally neutralized by the endogenous antioxidant system, including GSH, SOD, CAT, and GPx. Oxidative stress will happen with an increase in MPO, MDA, and NO levels along with dramatic decreases in the antioxidant system [68].

Many trials have shown that antioxidant therapy with natural products can be efficient in IBD. Antioxidant defenses can limit the negative outcomes that result from excessive ROS production. Antioxidant therapy significantly suppressed NF‐*κ*B p65 activation, lowered inflammatory cytokine levels, and demonstrated antioxidant effects [69–71]. By way of illustration, topical of *Elaeagnus angustifolia* hydroalcoholic extract as a natural antioxidant even at 2 days after treatment return tissue MDA, SOD, and MPO level to normal conditions [72]. Also, Honey *and Spirulina Platensis* reduced IL-6, TNF-*α*, and IL-1*β* and normalized MPO, NO, MDA, and PGE2 marker levels in colon tissues [73].

*Aloe vera* as a powerful antioxidant contains high amounts of anthraquinones such as barbaloin, emodin, and anthranol. Anthraquinones possess strong anti-inflammatory effects that can act as antioxidants and are involved in free-radical-mediated reactions during the inflammatory response to inhibit free radical-mediated cytotoxicity and lipid peroxidation [19]. Also, lophenol and cycloartanol as phytosterols found in *Aloe* can induce the downregulation of fatty acid synthesis involved in lipid peroxidations. It has been reported that *Aloe vera* can inhibit the inflammatory process by the reduction of leukocytes adhesion, inhibiting the cyclooxygenase pathways, and reducing prostaglandin E2 production [23]. Duansak reported that *Aloe vera* was characterized by the reduction of leukocyte adhesion, as well as proinflammatory cytokines, so the levels of TNF‐*α* and IL‐6 were also decreased significantly [74].

Reports also have shown that, in an acute IBD, anthraquinones inhibit the activation of NF‐*κ*B, JNK, p38 MAPK, Erk1/2, and 5‐LOX and suppress the expression of TNF‐*α*, IL‐6, IL‐1*β*, MPO, MDA, CINC‐1, MIP‐2, ICAM‐1, and MMP‐9. Considering these facts, natural anthraquinone derivatives have shown to exhibit potential therapeutic effects in the treatment and/or prevention of various inflammatory disorders [75]. Aloin, in the gel, is metabolized by the colonic flora to reactive *Aloe*-emodin, downregulating MMP-2/9, RAS, and vascular endothelial growth factor (VEGF) *via* reducing the DNA binding activity of NF-K*β* [23]. In another research article, the therapeutic influence of the *Aloe vera* on UC could be due to its beneficial properties such as anti-inflammatory effects or cell growth increment [76]. In a double-blind placebo-controlled study in 2004, Langmead et al. showed the beneficial effect of 4 weeks oral *Aloe vera* treatment for patients with mild-to-moderate UC (30% vs. 7% for placebo, respectively) [23]. However, the level of biochemical factors and its ethical concern due to the placebo control group were not evaluated.

Our study found that TNF-a, IL-6, NO, MPO, and MPA levels increased in the colitis group and decreased after antioxidant therapy. This increase in the colitis group may be explained by the response against oxidative stress. Biochemical results of the *Aloe vera*-treated group rectally as a powerful antioxidant were comparable with sulfasalazine. It would be interesting to note that 400 mg/kg *Aloe vera* extract rectally improved rats' general condition and mucosal and epithelial architecture, decreased cytokines, diminished inflammatory cell infiltration, reduced lamina propria edema, and led to thickening, which endorse both local and systemic effects of this extract on UC. In detail, the weight/length of the colon in the model group received 400 mg/kg *Aloe vera* extract rectally was significantly improved not only compared with the TNBS-control group (*P* < 0.001) but also compared with 200 mg/kg *Aloe vera* extract, rectally (*P* < 0.001), 200 mg/kg *Aloe vera* extract orally (*P* < 0.001), and 400 mg/kg *Aloe vera* extract, orally (*P* < 0.02). A similar pattern with lesser effects was seen in bodyweight assessments. The results showed that 400 mg/kg *Aloe vera* extract rectally could effectively protect rats from colitis caused by tissue damage. The MPO and MDA levels were significantly decreased after the administration of 400 mg/kg *Aloe vera* extract rectally compared to 200 mg/kg, rectally, 200 and 400 mg/kg, oral *Aloe vera* extract with *P* < 0.001 compared to all groups (Figure 4). Also, high levels of NO are associated with inflammation. Biological experiments confirmed that 400 mg/kg *Aloe vera* extract rectally can effectively reduce the NO compared to 200 mg/kg (*P* < 0.001) and 400 mg/kg (*P* < 0.001) oral *Aloe vera* extract. Also, 400 mg/kg *Aloe vera* extract rectally tended to decrease the NO level more compared to 200 mg/kg, rectally; however, the difference was not significant (*P* < 0.28). In the case of TNF-*α* and IL-6, the administration of *Aloe vera* extracts reduced these inflammatory cytokines with more reduction in group 6 compared to other treated groups although no significant differences were observed.

## 5. Conclusions

Current findings indicated that the administration of 400 mg/kg *Aloe vera* extract rectally on experimental colitis in rats can significantly attenuate the increased levels of TNF-a, IL-6, NO, MPO, and MPA and reduce the inflammations as compared to the TNBS-induced colitis model. The efficacy of *Aloe vera* extract is confirmed *via* mucosal healing, increased colon weight/length ratio, histopathological parameters, and restoring general conditions by increasing weight. These data could contribute to the formulation of therapeutic products of *Aloe vera* extract on IBD patients.

## Figures and Tables

**Table 1:** Criteria for scoring of gross morphologic damage [4].

Score	Gross morphology
0	No damage
1	Localized hyperemia, but no ulcers or erosions
2	Ulcers or erosions with no significant inflammation
3	Ulcers or erosions with inflammation at one site
4	Two or more sites of ulceration and/or inflammation
5	Two or more major sites of inflammation and ulceration or one major site of inflammation and ulceration extending >1 cm along the length of the colon

**Table 2:** Criteria for histological scoring of damage.

Appearance	Score
Loss of mucosal architecture	0, 1, 2, or 3 (absent, mild, or 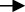 severe)
Cellular infiltration	0, 1, 2, or 3 (absent, mild, or 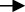 extensive)
Muscle thickening	0, 1, 2, or 3 (absent, mild, or 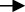 extensive)
Crypt abscess formation	0 or 1 (absent or present)
Goblet cell depletion	0 or 1 (absent or present)
Total	Summation

**Table 3:** The effects of different doses and routes of *Aloe vera* extracts and sulfasalazine administration.

Group	A: macroscopic score	B: histological score
Control	0	0.61 ± 0.8
TNBS colitis	5	10.09 ± 0.54
TNBS colitis plus *Aloe vera* extract 200 mg/kg intragastrically	3	8.30 ± 0.39
TNBS colitis plus *Aloe vera* extract 400 mg/kg intragastrically	2	6.81 ± 0.19
TNBS colitis plus *Aloe vera* extract 200 mg/kg rectally	2	7.19 ± 0.26
TNBS colitis plus *Aloe vera* extract 400 mg/kg rectally	1	6.85 ± 0.5
TNBS colitis plus *s*ulfasalazine intragastrically	1	6.44 ± 0.11

## Data Availability

The datasets used and analyzed during the current study are available from the corresponding author on reasonable request. All data are presented in the form of tables and figures.

## Abbreviations

UC: Ulcerative colitis
IBD: Inflammatory bowel diseases
ARRIVE: Animal Research Reporting In Vivo Experiments guidelines
IL-2: Interleukin-2
IL-6: Interleukin-6
IFN-*γ*: Interferon-*γ*
TNF-*α*: Tumor necrosis factor
MPO: Myeloperoxidase
MDA: Malondialdehyde
TBA: Thiobarbituric acid
ROS: Reactive oxygen species
RNS: Reactive nitrogen species
NO: Nitric oxide
HETAB: Hexadecyltrimethyl ammonium bromide
EDTA: Ethylenediaminetetraacetic acid.
